# Structured Rehabilitation Program for Multidirectional Shoulder Instability in a Patient with Ehlers-Danlos Syndrome

**DOI:** 10.1155/2020/8507929

**Published:** 2020-02-01

**Authors:** Takashi Kitagawa, Nobumasa Matsui, Dai Nakaizumi

**Affiliations:** ^1^Department of Physical Therapy, School of Health Sciences, Shinshu University, 3-1-1 Asahi, Matsumoto, Nagano 390-8621, Japan; ^2^Department of Rehabilitation, Japanese Red Cross Kanazawa Hospital, Kanazawa, Japan

## Abstract

Patients with Ehlers-Danlos syndrome (EDS) present many musculoskeletal disorders. The purpose of this case report was to describe the effectiveness of a scapular motor control program for a patient with multidirectional severe shoulder instability due to EDS, with 6-month follow-up. The patient was a 14-year-old female with EDS hypermobile type who suffered recurrent shoulder dislocation. Her chief complaints were bilateral shoulder discomfort and instability during writing motion. In the early part of intervention, she was prescribed exercise therapy for multidirectional instability (MDI) with orthosis. In the latter part of intervention, she was instructed in the scapular motor control program. Active and passive range of motion (ROM), sulcus sign, and Rowe score for shoulder instability were measured at baseline and at 3, 6, and 12 months after interventions. The shoulder ROM and instability score were improved after 6-month intervention. The findings from this report indicate that the scapular motor control program for shoulder instability would be effective even for patients with EDS hypermobile type. A patient who could not increase passive ROM due to dislocation is also able to achieve fair function of the shoulder joint instead of increasing active ROM. These positive outcomes indicate the possibility of benefit from the scapular motor control program for an MDI patient with EDS as a conservative treatment.

## 1. Introduction

The Ehlers-Danlos syndrome (EDS) is a connective tissue disorder that presents as skin extensibility, tissue fragility, and articular hypermobility [1]. EDS has been described as having 13 subtypes [2]. EDS classical types typically present skin hypermobility and joint hypermobility [3]. The physical activity level and quality of life of patients with EDS are affected by the severe musculoskeletal symptoms [4]. The younger generation typically complains of pain in the knee, back, and shoulder [5]. One of the severe musculoskeletal disorders is shoulder instability [4]. According to a systematic review of previous research, there are still no high-quality studies on hypermobile EDS physical therapy [6].

Recently, the results from a high-quality randomized control trial showed that there has been an established stage-based rehabilitation program for multidirectional instability (MDI) to achieve better shoulder function with the usual treatment [7]. However, patients with EDS were excluded from the study. There are few studies that have investigated rehabilitation for patients with shoulder instability due to EDS. The purpose of this case report was to describe the possibility of the effectiveness of a stage-based exercise program for a young female patient with multidirectional severe shoulder instability due to EDS with 6-month follow-up. The AB design was incorporated to compare the usual conservative treatment and the stage-based program.

## 2. Case Description

### 2.1. Participant History

The subject was a 14-year-old female patient with EDS hypermobile type. She had perceived her recurrent shoulder dislocation from childhood. She complained of shoulder pain and restriction of activity in daily life (ADL) during shoulder movement due to shoulder instability. There was no abnormal finding from radiological examination. There were no psychiatric diseases. She could dislocate her bilateral shoulder joint voluntarily. Her chief complaint was the bilateral shoulder discomfort and instability during writing motion. On her first examination by a doctor, she was treated conservatively, using the exercise program for 6 months [8]. On her second examination by a doctor at another hospital, she was also treated conservatively, adding an orthosis for MDI to the exercise program (intervention A) [9]. The patient received an explanation about this report and provided informed consent that complied with the Declaration of Helsinki.

### 2.2. Examination

The patient had a slight build with a height of 156 cm and weight of 44 kg. To evaluate her bilateral shoulder function, her active range of motion (ROM) (Table 1), sulcus sign, and shoulder instability in each direction were measured on the time point at the beginning of intervention A. Her passive ROM was not able to be measured because her humeral head was dislocated so easily with passive ROM exercise. Muscle strength was also difficult to measure due to dislocation with ease. Although her humeral head dislocated many times during rehabilitation, the doctor allowed her rehabilitation because there was no increase of persistent pain and she could centralize her humeral head voluntarily. The modified Rowe score was measured to evaluate shoulder instability [10] (Table 2).

### 2.3. Prognosis

EDS patients usually suffer from many musculoskeletal symptoms, even when they grow up [4]. On the other hand, it has been demonstrated that patients who have shoulder MDI will recover shoulder function during movement after rehabilitation programs [7, 9]. However, the prognosis of this patient was challenging because patients with EDS were excluded from the rehabilitation programs.

### 2.4. Intervention

The AB design includes some measurement of outcome variables throughout a baseline control/comparison phase A and then throughout another intervention phase B [9]. In the early part of rehabilitation, the patient was provided with an exercise program for MDI with orthosis [9] (intervention A). The term was continued for 3 months. In summary, she performed shoulder isometric muscle exercises using the TheraBand (Hygenic, Akron, Ohio) for the rotator muscles and scapula stabilizers with supervision by a physical therapist. The program includes five exercises: resistance exercises of shoulder abduction, internal rotation, external rotation, extension, and flexion. Assistance from a physical therapist to stabilize the humeral head was often needed so as not to dislocate it. The resistance during exercise was gradually increased according to her performance regarding shoulder dislocation. A physical therapy session was provided one to two times a week. Each session was 40 minutes. Compliance of home exercises was no more than about twice a week.

In the latter part of rehabilitation, the patient was instructed to perform the Watson program for MDI [7, 11, 12] (intervention B). The Watson program is an exercise program that aims for coordination of scapular muscles with strengthening rotator cuff and deltoid muscle. This phase also lasted for 3 months. In summary, this program consists of six stages that focus on retraining specific scapular control before any deltoid and rotator cuff exercise. The patient should make progress with exercise into functional and sport-specific ranges. She also received a physical therapy session one to two times a week. Each session was 40 minutes. In the first stage, correction of her humeral head by a physical therapist was effective during shoulder movement. The scapular control exercise was performed with assistance. She could make progress to stage 2 within 1 month. She could finally perform stage 5 exercises after 2 more months of rehabilitation. Compliance of home exercises was more than 3 days a week.

### 2.5. Outcome

The intervention was performed from July 2017 to January 2018. The patient was followed up until July 2018. The active ROM increased, while the passive ROM was not increased, due to easy dislocation (Table 1). The sulcus sign was still positive after intervention and at the time point of the final evaluation. The shoulder instability score was improved after intervention B (Table 2). She felt no pain and discomfort during writing motion after the time point. She could also control both humeral heads within proper position during ADL. A fair outcome was achieved by the final evaluation.

## 3. Discussion

The results from this case report are significant for the conservative treatment of MDI due to EDS. This AB-designed case report revealed two findings. First, the Watson program for shoulder instability could be effective even for patients with EDS hypermobile type. Second, a patient who could not increase passive ROM due to dislocation is able to achieve fair function of a shoulder joint, instead increasing active ROM.

Only one report has shown the effectiveness of physical therapy for the patient with EDS hypermobile type [13]. The importance of rehabilitation for patients with MDI is to achieve dynamic stability during their ADL and/or sports activities. Achieving anatomical stability (static stability) will be difficult even if a patient is treated operatively, so functional improvement is the primary target in rehabilitation. In other words, it is important to reinforce the cooperation of the muscles around the shoulder joint to keep the humeral head in the proper position during shoulder movement.

The traditional program in intervention A did not include functional training for the shoulder joint. It was aimed to reinforce muscle strength around the shoulder joint [8]. In contrast, the Watson program includes plyometric exercise, functional training, and movement-specific training [7]. The latter program and concept would have a better effect on this patient, in order to acquire dynamic stability of the shoulder joint during movement.

Another finding from this case report was that shoulder function achieved a fair level even though passive ROM was not increased after intervention. In other words, not static but dynamic stability is essential to achieve normal level of function through the cooperation of shoulder muscles. For some patients who failed through conservative management, surgery should be considered [14]. However, the best treatment for MDI has still been argued [15].

One of the purposes of each treatment for MDI patients is to regain normal kinetics during shoulder movement. Although anatomical reconstruction is difficult through conservative treatment, it is possible to achieve functional recovery through rehabilitation programs. In physical therapy for patients with typical shoulder injury or disease, a physical therapist usually should aim to expand passive ROM before active ROM. According to the insights from this case report, expanding passive ROM without dislocation would be difficult, and it is more important to expand active ROM with functional training for patients with MDI. It will be helpful to stabilize the glenohumeral joint during shoulder movement, focusing not only on muscle strengthening exercises but also on neuromuscular training.

This case report could not confirm the effectiveness for achieving fair function of a shoulder joint by the Watson program for MDI because an AB-designed case report was employed. Further case series should be done to investigate the effectiveness of this kind of intervention. ABA-designed reports are also needed because the design (control phase comes again after AB phase) would reveal the long-term effect of this intervention. In conclusion, the positive outcomes in this case report indicate the possibility of benefit from the Watson program for MDI patients with EDS as a conservative treatment.

## Figures and Tables

**Table 1:** Range of motion of shoulder joint on each time point.

	Baseline	After intervention A (3-month follow-up exam)	After intervention B (6-month follow-up exam)	Final evaluation (12-month follow-up exam)
Active flexion	30/35	45/45	155/150	160/160
Abduction	45/45	45/45	115/110	125/120
Passive flexion	25/30	35/30	35/30	35/35
Abduction	30/35	40/40	45/45	40/45

**Table 2:** The Rowe scores on each time point.

	Baseline	After intervention A (3-month follow-up exam)	After intervention B (6-month follow-up exam)	Final evaluation (12-month follow-up exam)
Stability score	0	0	30	10
Motion score	0	0	15	15
Function score	0	10	25	25
Total score	0	10	70	50
